# Suppression of RAF/MEK or PI3K synergizes cytotoxicity of receptor tyrosine kinase inhibitors in glioma tumor-initiating cells

**DOI:** 10.1186/s12967-016-0803-2

**Published:** 2016-02-09

**Authors:** Takashi Shingu, Lindsay Holmes, Verlene Henry, Qianghu Wang, Khatri Latha, Anupama E. Gururaj, Laura A. Gibson, Tiffany Doucette, Frederick F. Lang, Ganesh Rao, Liang Yuan, Erik P. Sulman, Nicholas P. Farrell, Waldemar Priebe, Kenneth R. Hess, Yaoqi A. Wang, Jian Hu, Oliver Bögler

**Affiliations:** Department of Neurosurgery, The University of Texas M. D. Anderson Cancer Center, 1515 Holcombe Boulevard, Houston, TX 77030 USA; Department of Cancer Biology, The University of Texas M. D. Anderson Cancer Center, 1881 East Road, Houston, TX 77054 USA; Department of Bioinformatics and Computational Biology, The University of Texas M. D. Anderson Cancer Center, 1881 East Road, Houston, TX 77054 USA; Department of Radiation Oncology, The University of Texas M. D. Anderson Cancer Center, 1515 Holcombe Boulevard, Houston, TX 77030 USA; Department of Chemistry, Virginia Commonwealth University, 901 West Franklin Street, Richmond, VA 23284-9005 USA; Department of Experimental Therapeutics, The University of Texas M. D. Anderson Cancer Center, 1881 East Road, Houston, TX 77054 USA; Department of Biostatistics, The University of Texas M. D. Anderson Cancer Center, 1515 Holcombe Boulevard, Houston, TX 77030 USA; Baylor College of Medicine, The University of Texas M. D. Anderson Cancer Center, Houston, USA; The University of Texas M. D. Anderson Cancer Center, 7007 Bertner Ave., Houston, TX 77030 USA

**Keywords:** Glioma, Stem cells, 3-D culture, Combination therapy, Synergistic, Autophagy, Receptor tyrosine kinase, Gompertz

## Abstract

**Background:**

The majority of glioblastomas have aberrant receptor tyrosine kinase (RTK)/RAS/phosphoinositide 3 kinase (PI3K) signaling pathways and malignant glioma cells are thought to be addicted to these signaling pathways for their survival and proliferation. However, recent studies suggest that monotherapies or inappropriate combination therapies using the molecular targeted drugs have limited efficacy possibly because of tumor heterogeneities, signaling redundancy and crosstalk in intracellular signaling network, indicating necessity of rationale and methods for efficient personalized combination treatments. Here, we evaluated the growth of colonies obtained from glioma tumor-initiating cells (GICs) derived from glioma sphere culture (GSC) in agarose and examined the effects of combination treatments on GICs using targeted drugs that affect the signaling pathways to which most glioma cells are addicted.

**Methods:**

Human GICs were cultured in agarose and treated with inhibitors of RTKs, non-receptor kinases or transcription factors. The colony number and volume were analyzed using a colony counter, and Chou-Talalay combination indices were evaluated. Autophagy and apoptosis were also analyzed. Phosphorylation of proteins was evaluated by reverse phase protein array and immunoblotting.

**Results:**

Increases of colony number and volume in agarose correlated with the Gompertz function. GICs showed diverse drug sensitivity, but inhibitions of RTK and RAF/MEK or PI3K by combinations such as EGFR inhibitor and MEK inhibitor, sorafenib and U0126, erlotinib and BKM120, and EGFR inhibitor and sorafenib showed synergy in different subtypes of GICs. Combination of erlotinib and sorafenib, synergistic in GSC11, induced apoptosis and autophagic cell death associated with suppressed Akt and ERK signaling pathways and decreased nuclear PKM2 and β-catenin in vitro, and tended to improve survival of nude mice bearing GSC11 brain tumor. Reverse phase protein array analysis of the synergistic treatment indicated involvement of not only MEK and PI3K signaling pathways but also others associated with glucose metabolism, fatty acid metabolism, gene transcription, histone methylation, iron transport, stress response, cell cycle, and apoptosis.

**Conclusion:**

Inhibiting RTK and RAF/MEK or PI3K could induce synergistic cytotoxicity but personalization is necessary. Examining colonies in agarose initiated by GICs from each patient may be useful for drug sensitivity testing in personalized cancer therapy.

**Electronic supplementary material:**

The online version of this article (doi:10.1186/s12967-016-0803-2) contains supplementary material, which is available to authorized users.

## Background

Malignant gliomas, including anaplastic astrocytoma and glioblastoma, are common primary tumors of the central nervous system, and characterized by aggressive cellular proliferation, diffuse infiltration, and resistance to cell death [1]. Diffuse infiltration of malignant glioma cells into adjacent brain parenchyma makes gross total removal by surgery difficult. Glioma cells that are resistant to cell death-inducing stimuli cause recurrence after radiation therapy and chemotherapy. For these reasons, the outcome of patients with malignant gliomas is poor [1]. The tumorigenesis of malignant gliomas has been associated with a number of alterations in related genes causing functional loss of tumor suppressors including PTEN, p53, NF1, RB1 or p16 proteins, and hyperfunction of oncogenic proteins such as EGFR, cyclin D1/3, E3 ubiquitin-protein ligase Mdm2, Mdm4, Met, Bcl-2, cyclin dependent kinase 4/6, PDGFRA and PI3K [2]. Among them, alterations of EGFR including gene amplification and activation mutations are observed in 40–57 % of malignant gliomas [2, 3]. The Cancer Genome Atlas (TCGA) Study also indicates that the vast majority of glioblastomas have aberrant RTK/RAS/PI3K signaling pathways [3]. Therefore, malignant glioma cells are thought to be addictive to these aberrant signaling pathways for their survival and proliferation, and thus targeting these signaling pathways has been thought to be effective on therapy of malignant gliomas [3]. However, a large number of clinical trials in which the targeted therapies were examined for malignant glioma patients indicate that monotherapies using single anticancer agents would have limited efficacy [4]. Recent studies indicate that inter- and intratumoral heterogeneities are major obstacles to the efficient targeted therapies for solid tumors [2–5]. In addition, key signaling nodes to which multiple oncogenic signaling pathways converge might not be inhibited sufficiently by monotherapies or even by combination therapies because of unexpected redundancy or alterations of feedback in the signaling pathway network with complex crosstalk [6–8]. Therefore, it is necessary to find rationale of effective combination treatments and to optimize therapies to individual patients.

Glioma tumor-initiating cells (GICs) are thought to play a critical role in initiation, regrowth and recurrence of the tumor [1, 9]. Therefore, GICs need to be considered a target of glioma therapy, and it might be essential to evaluate effects of therapies on GICs from individual patients in order to predict the clinical relevance of the treatments and optimize therapy to the patients. *In vitro* screening of anticancer therapy has been done mainly by clonogenic assay because the effect of the therapy on clonogenicity of the tumor cells is thought to be associated with the clinical therapeutic efficacy [10]. However, clonogenic assay using GICs has been a challenge because GICs aggregate in the stem cell culture media, and evaluation of the accurate tumor neurosphere/colony number requires single cell culture system or semi-solid matrix to prevent cell/colony aggregation. Single cell culture systems need large numbers of wells/plates and are not well suited for high-throughput screening of combination therapies [11]. Although colony formation assays of GICs or neural stem cells using gels have been reported, the growth of the colonies initiated by these cells in soft agar has not yet been well characterized [12–15]. In addition, a recent study suggested that proliferating cells with limited self-renewal capacity are more tumorigenic than glioma stem-like cells and thus therapeutic effects on these proliferating cells might be a better predictor for the in vivo efficacy [16]. Therefore, in drug sensitivity testing of gliomas, method by which we can evaluate both clonogenicity of GICs and cell proliferation of GICs and their descendant cells may be useful.

In this study, we cultured GICs in agarose and evaluated the number and volume of the colonies that reflect clonogenicity and cell proliferation, respectively, using a colony counter GelCount. With this method, we examined efficiency of combination treatments using RTK inhibitors, non-receptor kinase inhibitors and transcription factor inhibitors that affect the signaling pathways to which most glioma cells are thought to be addicted.

## Methods

### Antibodies and reagents

Erlotinib, lapatinib and sorafenib were purchased from LC laboratories (Woburn, MA), BKM120 was from Novartis (Basel, Switzerland), PD98059 and PP2 were from Selleck Chemicals (Houston, TX), U0126 and 3-methyladenine (3-MA) were from Sigma-Aldrich (St. Louis, MO), c-Myc inhibitor II was from EMD Millipore Corporation (Billerica, MA). Imatinib mesylate was generously provided from Novartis. A polynuclear platinum BBR3610 was synthesized by Dr. Nicholas P Farrelle (Virginia Commonwealth University) [17]. WP1066, an inhibitor of tyrosine phosphorylated STAT3 and STAT5 was synthesized by Dr. Waldemar Priebe (The University of Texas MD Anderson Cancer Center) [18]. These reagents except for 3-MA, BBR3610 and imatinib were dissolved in DMSO (Sigma-Aldrich). 3-MA was dissolved in culture media, and imatinib and BBR3610 were dissolved in PBS. Antibodies for Akt, AMPK, Atg5, Bad, c-Myc, EGFR, ERK, Met, poly-ADP ribose polymerase (PARP), pyruvate kinase isozyme M2 (PKM2), and ribosomal protein S6, or phosphorylated forms of Akt (Ser473), AMPK (Thr172), Bad (Ser136), EGFR (Tyr1173), ERK (Thr202/Tyr204), Met (Tyr1234/1235), and S6 (Ser235/236) were obtained from Cell Signaling Technology, Inc. (Danvers, MA). Antibodies for Bcl-2, Bcl-XL, β-catenin, Mcl-1, p53, and PTEN were obtained from Santa Cruz Biotechnology, Inc. (Santa Cruz, CA). Anti-LC3B antibody was obtained from Novus Biologicals, Inc. (Littleton, CO). Antibody for CD133 was obtained from Abcam plc (Cambridge, UK). Antibodies for lamin B and nestin were obtained from EMD Millipore. Antibodies for β-actin and vinculin were from Sigma-Aldrich.

### Cell lines

Human malignant glioma cell line U87-MG was from American Type Culture Collection (Manassas, VA), and human malignant glioma cell lines LNZ308 and LN428 were from Dr. Nicolas de Tribolet (Lausanne, Switzerland), during 2003–2007. These cells were maintained in DMEM (Life Technologies Corporation, Carlsbad, CA) containing 10 % FBS (Life Technologies), 100 units/ml penicillin G, 100 μg/ml streptomycin, and 50 mg/ml l-glutamine (Life Technologies) in a humidified atmosphere containing 7 % CO_2_ at 37 °C. Human glioma tumor-initiating cells derived from glioma sphere culture were established by Dr. Frederick F Lang (The University of Texas MD Anderson Cancer Center) as described previously, in which GSC11 and GSC47 cells are non-mesenchymal subtype and GSC2 and GSC20 cells are mesenchymal subtype [19, 20]. These cells are maintained in DMEM/Ham’s F12 medium (F12) (Life Technologies) containing 1× B27 supplement (Life Technologies), 20 ng/ml EGF and 20 ng/ml basic FGF (Life Technologies), 100 units/ml penicillin G, 100 μg/ml streptomycin, and 50 mg/ml l-glutamine in a humidified atmosphere containing 7 % CO_2_ at 37 °C. Although the authentication of the purchased cell lines was not done by authors, DNA fingerprinting was done for testing cell line contamination using GenomeLab Human STR Primer Set according to the manufacturer’s instruction (Beckman Coulter, Brea, CA). Infection by mycoplasma was examined by Mycoalert (Lonza, Basel, Switzerland) and infected cells were treated with BM cyclin (Roche Diagnostics, Indianapolis, IN).

## 3D cell culture in agarose gel

For analyses of number and volume (total biomass) of tumor spheres in agarose gel, culture media containing 0.7 % agarose (Thermo Fisher Scientific, Waltham, MA) was used as the bottom layer, and culture media containing 0.4–0.5 % agarose and tumor cells were placed in the middle layer, and then, culture media were added to the well in 96 or 24 well plates (Corning Incorporated, NY) [21]. Cells were seeded at 100, 150 and 200 cells in 96 well plate as triplicate, or 250 and 500 cells in 24 well plate as duplicate. The final volumes of the bottom, middle and top layers for 96 well plate and 24 well plate were 100, 100 and 50, and 500, 500, and 200 μl, respectively. After 4 h of seeding cells, culture media containing indicated reagents were added and cells were further incubated. Plates were scanned at the indicated time points in time course experiments or 12–14 days after addition of drugs in colony formation analysis, and number and total biomass of colonies larger than 40 μm in diameter were evaluated using GelCount colony counter system (Oxford Optronix Inc., UK).

### Analysis of tumor growth

Surviving fraction in colony formation analysis and total biomass in volume analysis were calculated by the methods reported by Franken and Kajiwara, respectively [10, 21]. In vivo tumor growth in xenograft models can be described using an equation of Gompertz model as: V = V_0_ exp[(A/α)(1–exp[–αt])] where V_0_ = initial volume, A and α = constant, V = volume at any time t [22]. In this study, colony number and total biomass were plotted against time and examined by non-linear regression analysis against Gompertz growth curve where V_0_ = initial colony number and initial total biomass, respectively. Coefficients of determination (R^2^) were calculated using GraphPad Prism software (GraphPad Software Inc., San Diego, CA). Combination indices of drugs in colony formation analysis were calculated by modified method of Chou and Talalay [23], and combination effect was thought to be synergistic when the combination index was less than 0.9. Tumor growth delay was calculated using the formula (Ti−C)/C where Ti and C were time (days) for treated and control tumors to reach double of total biomass at treatment, respectively [24].

### Immunoblotting

After treatment with reagents, cells were harvested, washed with PBS, lysed in 50 mM Tris–HCl (pH 7.5), 150 mM NaCl, 1 % Igepal-630, 1 mM EDTA, aprotinin, leupeptin, protease inhibitor and phosphatase inhibitor (Roche Diagnostics), and 1 mM PMSF and centrifuged at 10,000×*g* at 4 °C for 20 min. The supernatant was used as the cell lysate. Extraction of nuclear and cytoplasmic fractions of cells was done using Nuclear Extraction Kit (Sigma-Aldrich) according to the manufacture’s instruction. Ten-fifteen microgram of proteins were subjected to standard western blotting and the blots were quantified with ImageJ software (NIH, Bethesda, MD).

### Survival analysis of nude mice with intracranial tumor

The intracranial tumor model was made using male nude mice (Experimental Radiation Oncology at M. D. Anderson Cancer Center) as described previously [25]. Through the screw guide, at a depth of 3.5 mm from the skull, 5 μl aliquots of 5 × 10^5^ GICs in DMEM/F12 were inoculated at a rate of 1.0 μl/min. Mice were randomized into four groups (4–8 mice per each group) and treatment was initiated 1 week after tumor implantation. Erlotinib and sorafenib were dissolved in solvent, OraPlus (Paddock Laboratories Inc., Minneapolis, MN), and administered at doses of 50 mg/kg of erlotinib and 50 mg/kg of sorafenib orally three times a week for a total of twelve treatments. Mice of control group were treated with the solvent. All mouse studies were performed in the veterinary facilities of The University of Texas M. D. Anderson Cancer Center in accordance with IACUC, state, federal, and ethical regulations for experimental animal care.

### Cell viability assay

For trypan blue dye exclusion assay, tumor cells were seeded at 2 × 10^4^ cells per well in 24-well flat-bottomed plates and treated with reagents for indicated duration. Cells were dissociated by using accutase (Life Technologies) and the number of viable or dead cells (total >300 cells) was counted in at least three different fields.

### Quantification of apoptotic or non-apoptotic cell death with annexin V and propidium iodide

After treatment with indicated reagents, cells were stained with FITC-conjugated annexin V and propidium iodide for 15 min according to the manufacturer’s instruction (BD Biosciences, San Jose, CA), and then analyzed with FACSCalibur (Becton, Dickinson and Company, Franklin Lakes, NJ) using Cell Quest Pro software (Becton, Dickinson and Company).

### Analysis of LC3 localization

A GFP-tagged LC3 (GFP-LC3) expression vector was kindly provided by Drs. N Mizushima (The University of Tokyo, Tokyo, Japan) and T Yoshimori (Osaka University, Suita, Japan) [26]. Tumor cells were seeded in 24 well plates and transfected with GFP-LC3 expression vector using FuGENE HD Transfection Reagent (Roche Diagnostics). Cells were incubated overnight and treated with indicated reagents for 8 h. Cells were dissociated by using accutase, fixed with 4 % paraformaldehyde, mounted onto glass slides with VECTASHIELD Mounting Medium (Vector Laboratories Inc., Burlingame, CA), and then examined using fluorescence microscope. The number of cells with or without GFP-LC3 punctate pattern (total >300 cells) was counted in at least three different fields.

### RNA interference

siRNA was synthesized by Thermo Fisher Scientific (Dharmacon). The sequence of the siRNA targeting Atg5 was CAACUUGUUUCACGCUAUAdTdT. siCONTROL Non-Targeting siRNA#2 was purchased from Thermo Fisher Scientific and used as control siRNA. Cells were transfected with siRNAs (3 μg) by electroporation using Nucleofector and a Cell Line Nucleofector^®^ Kit T (Amaxa Biosystems, Gaithersburg, MD) according to the manufacturer’s instruction. After incubated overnight, cells were harvested, dissociated by using accutase, and seeded in 24 well plates for cell viability assay or 6 well plates for immunoblotting. After 3–4 h at 37 °C, cells were treated with reagents and subjected to the indicated assays.

### Reverse phase protein array

Reverse phase protein array (RPPA) study was performed as described previously [7]. Briefly, serially diluted lysates were arrayed on nitrocellulose-coated slides (Grace Bio-Labs, Inc., Bend, OR) by Aushon 2470 Arrayer (Aushon BioSystems, Billerica, MA). Total 5808 array spots were arranged on each slide including the spots corresponding to positive and negative controls prepared from mixed cell lysates and dilution buffer, respectively. Each slide was probed with a validated primary antibody plus a biotin-conjugated secondary antibody. A panel of 171 antibodies with a Pearson correlation coefficient between RPPA and western blotting of greater than 0.7 were used in RPPA study. The signal obtained was amplified using a Dako Cytomation–catalyzed system (Dako, Glostrup, Denmark) and visualized by DAB colorimetric reaction. The slides were scanned, analyzed, and quantified using a customerized-software Microvigene (VigeneTech Inc., Carlisle, MA) to generate spot intensity. Each dilution curve was fitted with a logistic model (“Supercurve Fitting” developed by the Department of Bioinformatics and Computational Biology in The University of Texas MD Anderson Cancer Center, “http://bioinformatics.mdanderson.org/OOMPA”). Results were normalized, transformed to base 2 logarithms and median centered, and the data from RPPA with three replicate samples were analyzed using two-way analysis of variance with an interaction term to assess whether the combined effect was significantly different from the sum of the individual effects.

### Gene expression analysis

Paired-end whole transcriptome sequencing of GIC cultures was performed on the Illumina HiSeq platform after random priming and rRNA reduction. Each sample generated about 50 million paired-ends; each end was 75 bp in size. Short transcript reads were mapped to 20,010 human protein coding genes in Ensembl reference transcriptome (ENSEMBL version 64). Downstream data analyses and RPKM (reads per kilobase per million reads) values were generated using pipeline for RNA sequencing data analysis (PRADA, PMID: 24695405) by involving Burroughs-Wheeler alignment, Samtools, and Genome Analysis Toolkit.

### Statistical analysis

Paired Student’s *t* test (two-tailed) was used for comparison between two groups, and ANOVA with Tukey’s post hoc test was used for four groups. For the animal experiment, pairs of Kaplan–Meier survival curves were compared by the log-rank Mantel-Cox test using GraphPad Prism software. Differences were considered statistically significant when provided *P* was less than 0.05.

## Results

### Glioma cell growth in agarose

**Fig. 1:**
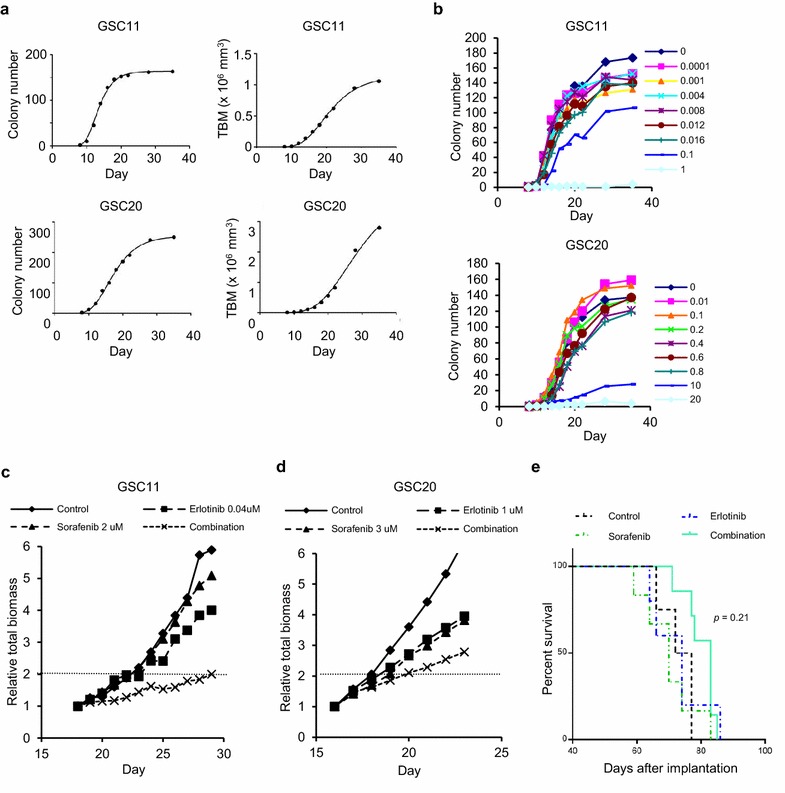
Growth of glioma tumor-initiating cells (GICs) in agarose and growth suppression of GICs in vitro and in vivo by drug treatments. **a** Number (*left panels*) and volume [total biomass (TBM), *right panels*] of colonies of GSC11 (*upper panels*) and GSC20 (*lower panels*) cells cultured in agarose were plotted against time and the growth curves were fitted to the Gompertzian curve. The coefficients of determination between actual and fitted data (R^2^) >0.99 in all panels. **b** GSC11 and GSC20 cells were seeded in agarose and treated with erlotinib at day 0, and the colony number was evaluated by using GelCount. Results shown are representative of three independent experiments. **c**, **d** GSC11 (**c**) and GSC20 (**d**) cells were seeded in agarose and treated with erlotinib and/or sorafenib after colony formation, and TBM was evaluated by using GelCount. Relative TBM to that at initiation of the treatment was plotted against time. *Broken lines* were for evaluation of doubling time to calculate tumor growth delay. Results shown are representative of three independent experiments done in duplicate. **e** Nude mice were inoculated with GSC11 cells, and after 1 week erlotinib and/or sorafenib were administered. The Kaplan–Meier survival curves were analyzed by log-rank test

The Gompertz function has been used for tumor growth analysis because in vivo and in vitro tumor growth correlates with the function [22, 27]. We evaluated correlation between the Gompertz function and increases in colony number or total volume of the colonies (total biomass, TBM) of glioma cells in 3-dimensional (3D) culture system using agarose. As shown in Fig. 1a, increases of colony number and TBM in GICs correlated with the Gompertz function where coefficients of determination were larger than 0.99. Each cell line examined has specific values of A and α of the Gompertz equation (Additional file 1: Table S1).

### Growth suppression of glioma cells by monotherapies and combination therapies

The TCGA study has revealed that 90 % of glioblastomas have aberrant signaling in RTK/RAS/PI3K signaling pathways and thus molecules in these signaling pathways have been thought to be therapeutic targets [3]. However, successful treatments targeting these signaling pathways remain to be clarified [5–8]. Considering multiplicity of cancerous signaling pathways with complex crosstalk in a tumor cell, targeting relatively upstream molecule such as RTK that could affect multiple oncogenic signaling pathways might be reasonable. To overcome signaling redundancy, combined inhibition of multiple molecules that upregulate the same signaling pathway might be necessary. Recent study suggests that inhibition of relatively distal downstream molecule such as mTOR causes activation of the upstream oncogenic molecule such as Akt by decreasing feedback signaling and thus reduces efficacy of erlotinib in the combination therapy [8]. Therefore, we hypothesized that effective combination therapies consist of inhibitors of relatively upstream molecules including cellular membrane receptors and their proximal downstream molecules, and then tested combination effects of RTK inhibitors and RAF/MEK or PI3K inhibitors.

At first, we confirmed dose dependent effects of erlotinib on colony formation in the agarose-based 3D culture system (Fig. 1b; Additional file 2: Figure S1A) and then evaluated IC_50_ values in monotherapies of reagents including RTK inhibitors erlotinib, lapatinib, and imatinib, and U0126 and BKM120 that suppress MEK and PI3K, respectively, in GICs with examination of phosphorylation or expression of EGFR, Met or PTEN protein or expression of genes related with RTKs, PI3K, RAS and p53/Rb pathway (Additional file 2: Figure S1B, Additional file 3; Table 1). Sorafenib is a multikinase inhibitor that suppresses VEGFR2, PDGFR, and RAF and thus was used as an RTK and RAF/MEK signaling inhibitor in this study. GSC11 cells, which are of the non-mesenchymal subtype, showed the highest sensitivity to EGFR inhibitors, though expression or phosphorylation of EGFR was not the highest in this line amongst the examined cell lines and PTEN was not detected (Additional file 2: Figure S1B; Table 1). GSC47 cells, which showed high EGFR expression and phosphorylation and intermediate levels of total and phophso-Met without PTEN (Additional file 2: Figure S1B) and are of the non-mesenchymal subtype, were relatively sensitive to erlotinib and lapatinib (Table 1). GSC20 cells, which had relatively low expression and phosphorylation levels of EGFR and low levels of total and phospho-Met and are of the mesenchymal subtype that is thought to be the most therapy-resistant subtype, showed relative resistance to EGFR inhibitors (Additional file 2: Figure S1B; Table 1) [3, 19]. GSC2 cells, which had high expression and phosphorylation levels of EGFR with PTEN expression and are of the mesenchymal subtype, also showed relative resistance to EGFR inhibitors (Additional file 2: Figure S1B; Table 1). In the presence of 10 % FBS, U87 cells, which have relatively high expression and low phosphorylation of EGFR, were also relatively resistant to EGFR inhibitors (Additional file 2: Figure S1B; Table 1). Therefore, it was difficult to predict sensitivity of glioma cells to EGFR inhibitors by status of EGFR, Met and PTEN despite previous encouraging reports, while cells with a mesenchymal profile tended to be more resistant to EGFR inhibitors than those with non-mesenchymal profile that is consistent with previous studies [28–32]. It was also difficult to predict the sensitivity of the GICs to the other treatments by subtype or the expression levels of genes listed in Additional file 3.

**Table 1:** IC_50_ values of drugs in glioma tumor-initiating cells and U87 cultured in agarose

Drugs	GSC11	GSC47	GSC20	GSC2	U87
Receptor tyrosine kinase inhibitors					
Erlotinib (μM)	0.11 ± 0.03	0.49 ± 0.15	0.82 ± 0.34	3.08 ± 0.55	3.72 ± 0.60
Lapatinib (μM)	0.64 ± 0.22	1.86 ± 0.16	4.39 ± 1.08	3.04 ± 0.99	7.01 ± 2.20
Sorafenib (μM)	2.59 ± 0.50	1.09 ± 0.44	2.21 ± 0.16	3.51 ± 0.25	5.24 ± 0.01
Imatinib (μM)	6.37 ± 0.90	NE	11.0 ± 1.57	6.28 ± 2.24	3.24 ± 2.10
Non-receptor kinase inhibitors					
U0126 (μM)	14.0 ± 4.72	18.0 ± 3.25	4.69 ± 1.33	11.0 ± 0.25	11.1 ± 5.35
BKM120 (μM)	0.41 ± 0.05	0.46 ± 0.21	0.36 ± 0.05	0.49 ± 0.10	0.59 ± 0.21
PP2 (μM)	1.93 ± 0.50	NE	6.39 ± 2.46	4.33 ± 0.34	NA > 20
Transcription factor inhibitors					
WP1066 (μM)	2.46 ± 0.62	NE	1.55 ± 0.13	1.96 ± 0.13	1.59 ± 0.40
Myc II (μM)	22.3 ± 3.57	NE	13.3 ± 1.50	19.0 ± 3.47	27.0 ± 5.68
DNA cross-linking agent					
BBR3610 (nM)	46.6 ± 23.0	NE	87.3 ± 33.5	117.3 ± 71	1.46 ± 1.26

Data are means ± 95 % confidence intervals of at least two independent experiments

*NE* Not examined, *NA* Not available, *Myc II* c-Myc inhibitor II

Next, cells were treated with combinations that inhibit RTK and RAF/MEK or PI3K, and combination indices at IC_50_ were calculated. To clarify the specific importance of RAF/MEK and PI3K in GICs, we also tested inhibitors of Src family kinases (PP2), STAT3/STAT5 (WP1066), and c-Myc (c-Myc inhibitor II) that are also related with RTK/RAS/PI3K signaling pathways, and a DNA cross-linking agent (BBR3610) as a different class of drug that does not target specific signaling molecules [17, 18]. We expectedly found synergistic effects in combinations that affect RTK and RAF/MEK or PI3K in multiple GIC lines (Table 2) while combinations of RTK inhibitors with a Src inhibitor, transcription factor inhibitors or DNA cross-linking agent were not synergistic (Additional file 1: Table S2). Lapatinib instead of erlotinib or PD98059 instead of U0126 reproduced synergistic effects in the combination treatments, confirming importance of inhibiting EGFR and MEK signaling pathways in the synergy (Table 2). We also examined combination effects of U0126 and inhibitors of downstream molecules in EGFR signaling in order to examine if the synergy is reproduced by inhibiting downstream molecules instead of EGFR inhibition in GSC20 (Additional file 1: Table S2). However, neither of the combinations of U0126 with BKM120, PP2, WP1066, or c-Myc inhibitor II was synergistic. In addition, combination of U0126 and BKM120 was not synergistic in either of cell lines (Additional file 1: Table S2). Taken together, it appears that combined suppression of RTK and its distal downstream molecule such as transcription factor or inhibition of two parallel signaling molecules such as MEK and PI3K are not sufficient for synergistic cytotoxicity. Instead combining an RTK inhibitor with a reagent that targets relatively proximal downstream molecules including RAF/MEK or PI3K induces synergistic antiglioma effects.

**Table 2:** Synergistic combinations of targeted drugs in glioma tumor-initiating cells

Drugs	GSC11	GSC47	GSC20	GSC2
Erlotinib + Sorafenib	0.85 ± 0.23	1.30 ± 0.16	1.21 ± 0.26	1.04 ± 0.24
Lapatinib + Sorafenib	0.82 ± 0.27	1.17 ± 0.40	1.18 ± 0.05	1.32 ± 0.06
Erlotinib + U0126	0.98 ± 0.24	2.14 ± 0.75	0.73 ± 0.08	0.52 ± 0.31
Erlotinib + PD98059	NE	NE	0.79 ± 0.17	NE
Lapatinib + U0126	NE	NE	0.86 ± 0.12	NE
Erlotinib + BKM120	1.16 ± 0.19	NE	1.04 ± 0.24	0.71 ± 0.05
Sorafenib + U0126	0.67 ± 0.13	0.73 ± 0.09	1.20 ± 0.59	0.56 ± 0.30

Chou and Talalay combination indices (CI) are shown as means ± 95 % confidence intervals of at least two independent experiments. Synergistic interactions were confirmed by three independent experiments. According to the original study by Chou and Talalay, CI <1, = 1; and >1 indicate synergistic, additive, and antagonistic, respectively. In this study, combination effect was thought to be synergistic when CI was less than 0.9

*NE* Not examined

Combinations of EGFR inhibitor with sorafenib were synergistic in GSC11 cells, which are of the non-mesenchymal subtype, but not synergistic in GSC47 cells, which are also of the non-mesenchymal subtype (Table 2). In addition, GSC2 and GSC20, which are both of the mesenchymal subtype, showed different sensitivities to combination therapies (Table 2). These results indicate that GICs show diverse sensitivity to therapies and that personalization is necessary for therapy even in the same subtype of glioma.

Next we evaluated the combined effect of erlotinib and sorafenib on the total biomass in time course study. Both tumor growth delay analysis and combination index at 1 week after treatment indicated synergistic effects in GSC11 cells, while tumor growth delay analysis showed synergy but combination index indicated only additivity in GSC20 cells (Fig. 1c, d; Additional file 1: Table S3). Considering these findings in total biomass analysis together with those from the colony formation study (Table 2), we concluded that GSC11 cells are sensitive to combination of erlotinib with sorafenib, and evaluated the effects of the combination therapy on nude mice bearing brain tumor (Fig. 1e). Although analyses of the survival curves and the median survival time did not indicate statistically significant difference, the median survival time of the combination group (83d) was remarkably improved by the combination treatment compared with those of control (74.5d), erlotinib (74d) and sorafenib (70d) groups, even with the doses that did not improve the survival in the monotherapies.

### Combination treatment-induced apoptotic and autophagic cell death

**Fig. 2:**
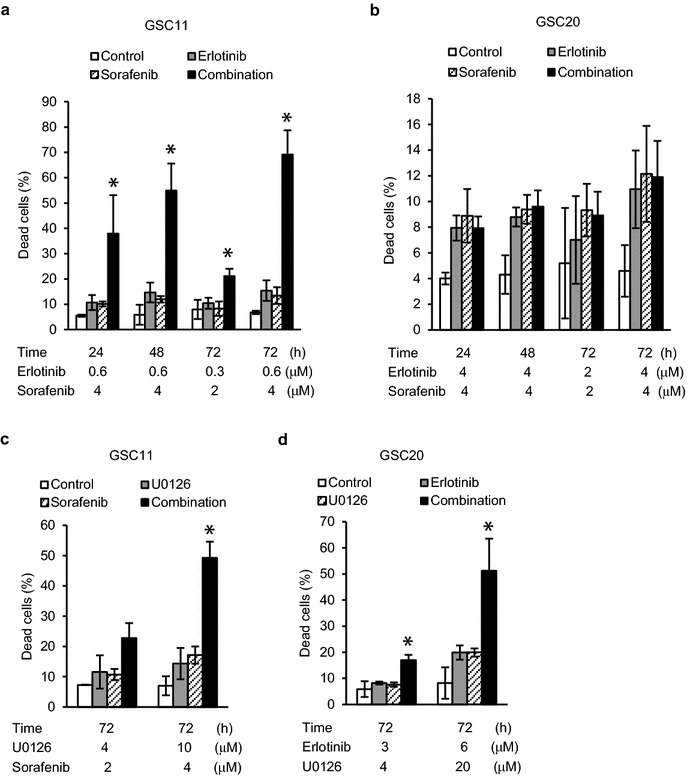
Increases of cell death by combination treatments in glioma tumor-initiating cells. GSC11 (**a** and **c**) and GSC20 (**b** and **d**) cells were treated with vehicle (control) or indicated drugs for indicated periods of time and analyzed with trypan blue dye exclusion assay to calculate percentage of dead cells. Data are expressed as the means of at least two independent experiments done in duplicate. *Error bars* are 95 % confidence intervals. *means *p* < 0.05 in ANOVA and Tukey’s post hoc test in comparison of combination treatment group with any of the other groups

**Fig. 3:**
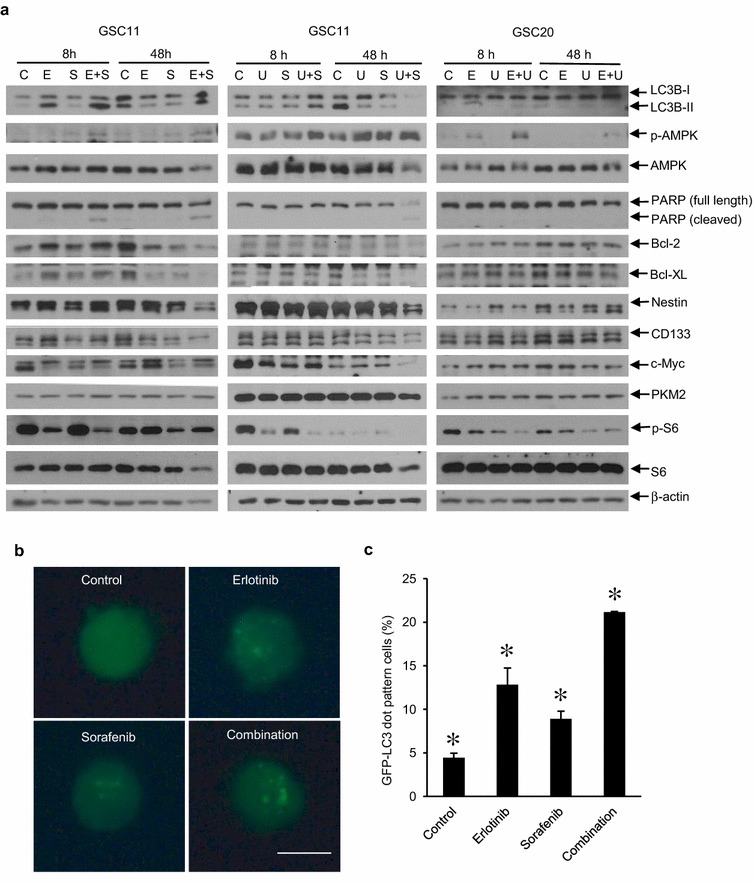
Induction of autophagy and apoptosis, and decrease of glioma stemness marker proteins by synergistic combination treatments in glioma tumor-initiating cells. **a** Cells were treated with vehicle as control (C), erlotinib (E), sorafenib (S), U0126 (U) and combinations for indicated duration, and 10 μg of extracted proteins in each sample were subjected to immunoblotting using indicated antibodies to detect total or phosphorylated (p-) proteins. Concentrations of drugs were as follows: erlotinib; 0.6 μM for GSC11 and 6 μM for GSC20, sorafenib; 4 μM for GSC11, U0126; 10 μM for GSC11 and 20 μM for GSC20. β-actin was examined as loading control. Results shown are representative of at least two independent experiments. **b** and **c** GSC11 cells with transfection of GFP-LC3 were treated with vehicle (control), 0.6 μM erlotinib, 4 μM sorafenib or the combination for 48 h. **b** Scale bar is 5 μm. **c** Cells with *punctate pattern* of GFP-LC3 were counted as autophagic cells. Data shown are the means of two independent experiments. *means significant difference (*p* < 0.05) between any two of the four groups by ANOVA and Tukey’s post hoc analysis

**Fig. 4:**
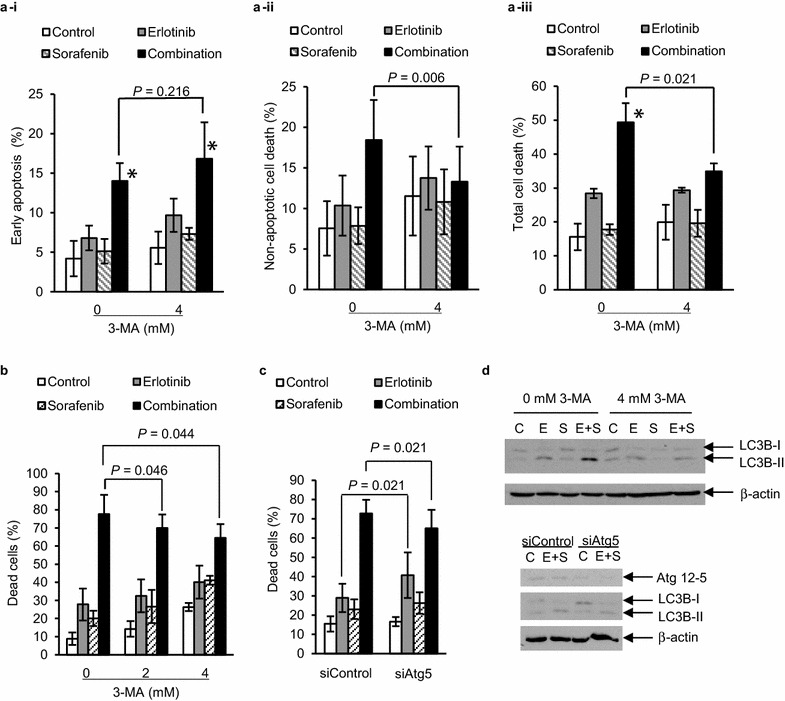
Decrease of combination therapy induced cell death by inhibiting autophagy in GSC11 cells. **a–d** GSC11 cells with 3-methyladenine (3-MA) treatment (**a**, **b** and *upper panel* of **d**) or with transfection of siRNA targeting Atg5 (siAtg5) (**c** and *lower panel* of **d**) were treated with vehicle (control), 0.6 μM erlotinib, 4 μM sorafenib or the combination for 8 h for immunoblotting using anti-LC3B or anti-Atg5 antibodies (**d**), or 72 h for apoptosis assay using propidium iodide (PI), annexin V (AV), and flow cytometer (a-i–a-iii ) or trypan blue dye exclusion assay (**b** and **c**). (a-i) Early apoptosis; PI (−) with AV (+). (a-ii) Non-apoptotic cell death; PI (+) with AV (−). (a-iii) Total cell death; PI (+). **a**–**c** Data are expressed as the means of three independent experiments. *means *p* < 0.05 in ANOVA and Tukey’s post hoc analysis comparing combination treatment group with others. *p* values shown were calculated by student’s paired *t* test. *Error bars* are 95 % confidence intervals. **d** Inhibitory effects of 3-MA and siAtg5 on LC3B-II increase was confirmed by immunoblotting. β-actin was examined as loading control. Results shown are representative of two independent experiments

Analysis of cell death revealed that combination treatment with erlotinib and sorafenib significantly increased cell death in GSC11 but not in GSC20 cells (Fig. 2a, b). Other synergistic combinations identified in the colony formation assay, such as U0126 and sorafenib in GSC11 or erlotinib and U0126 in GSC20, also increased cell death significantly (Fig. 2c, d). In immunoblotting, the synergistic combination treatments increased both microtubule-associated protein 1 light chain 3B-II (LC3B-II) and phosphorylated AMPK 8 h after treatment, and induced cleavage of PARP or decrease of full length PARP 48 h after treatment, indicative of autophagy and apoptosis, respectively (Fig. 3a). Those treatments also decreased anti-cell death proteins such as Bcl-2 and Bcl-XL (Fig. 3a). Increase of autophagic and apoptotic cells by combination of erlotinib with sorafenib was confirmed by transient GFP-LC3 expression experiments and annexin V staining, repectively (Figs. 3b, c and 4a). Inhibition of autophagy by 3-MA increased apoptotic cell death but decreased non-apoptotic and total cell death (Fig. 4a–c). Knockdown of Atg5 also decreased total cell death induced by the combination treatment (Fig. 4d). These results indicate that autophagy in synergistic combination therapies is cytokilling. We also examined CD133 and nestin that are thought to be marker proteins for glioma cell stemness (Fig. 3a) [9]. The synergistic combination treatments effectively reduced the amounts of those proteins 48 h after treatment. Taken together, it appears that the combination therapies induce synergistic cytotoxicity through apoptosis and autophagic cell death, and efficiently decrease glioma cells with stemness.

### Combination treatment-induced alterations in signaling pathways

**Fig. 5:**
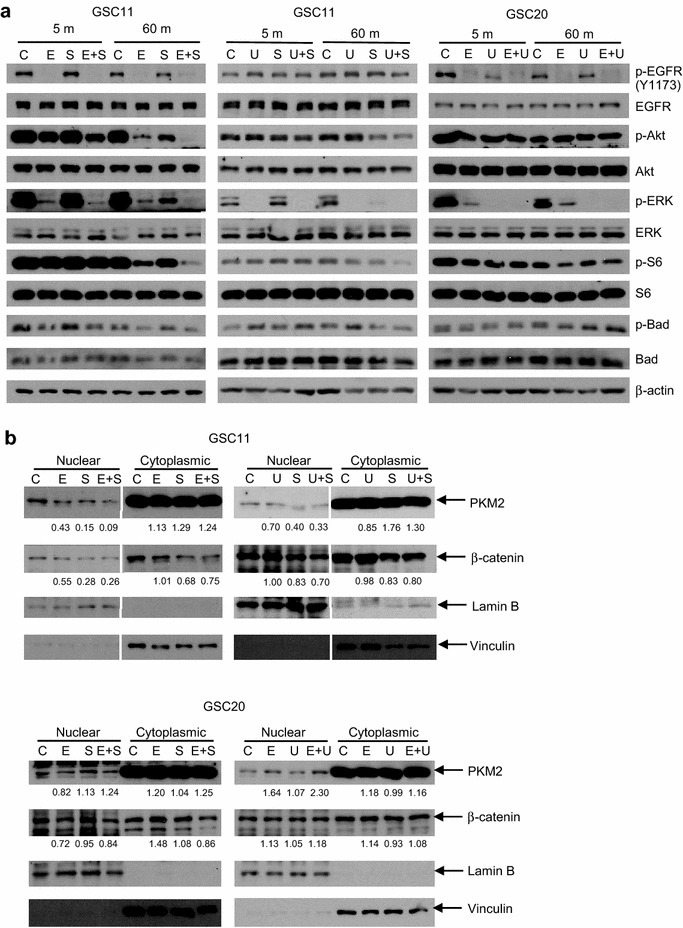
Alterations of signaling pathways by synergistic combination treatments in glioma tumor-initiating cells. **a** Cells were treated with vehicle as control (C), erlotinib (E), sorafenib (S), U0126 (U), and combinations for indicated duration, and 10 μg of extracted proteins in each sample were subjected to immunoblotting using indicated antibodies to detect total or phosphorylated (p-) proteins. Concentrations of drugs were as follows: erlotinib; 0.6 μM for GSC11 and 6 μM for GSC20, sorafenib; 4 μM for GSC11, U0126; 10 μM for GSC11 and 20 μM for GSC20. β-actin was examined as loading control. **b** GSC11 or GSC20 cells were treated with indicated reagents for 24 h and 10 g of extracted nuclear or cytoplasmic proteins in each sample were subjected to immunoblotting using anti-PKM2 and anti-β-catenin antibodies. Lamin B and vinculin were examined as loading controls of nuclear proteins and cytoplasmic proteins, respectively. Relative values of blots of PKM2 and β-catenin in treatment groups to control were calculated using ImageJ

We did reverse phase protein array (RPPA) analysis to examine alterations in a panel of 171 signaling related proteins (Additional files 4, 5) in GSC11 cells 60 min after treatment with erlotinib and sorafenib. As shown in Additional file 1: Table S4, proteins on which the combination treatment induced a statistically significant synergistic effect included phosphorylated proteins that are related with PI3K/Akt or RAS/MEK/ERK signaling pathways. In addition, 16 proteins that are not directly associated with downstream of PI3K/Akt or MEK/ERK signaling pathways were altered toward tumor suppressive direction, including proteins related with apoptosis (Bax, caspase 7), cell cycle (CDK1, Chk1, stathmin), fatty acid metabolism (SCD1), gene transcription (Smad, STAT5a, TTF1), glucose metabolism (G6PD, GAPDH), histone methylation (SETD2), iron transport (TFRC), stress response (NDRG1), or tyrosine kinase (c-Met, Lck) (Additional file 5). These results suggest that inhibition of RTK affects various cancerous signaling pathways that might not be reproduced by suppressing small number of downstream molecules even though they might be signal converging molecules. We confirmed remarkable decreases in phosphorylated forms of Akt, S6 and ERK in GSC11 cells 60 min after treatment with erlotinib and sorafenib in immunoblotting (Fig. 5a). Another synergistic combination therapy in GSC11 cells, U0126 and sorafenib, also inhibited phosphorylations of Akt and S6 (Fig. 5a). These combination treatments decreased nuclear PKM2 and β-catenin 24 h after treatment (Fig. 5b). However, combination of erlotinib with U0126, which was synergistic in GSC20, did not decrease phosphorylated Akt or S6 remarkably or nuclear PKM2 or β-catenin while phospho-ERK was efficiently decreased by U0126 alone in GSC20 cells (Fig. 5a, b). These results indicate that immediate inhibition in at least Akt signaling and subsequent decreases in nuclear PKM2 and β-catenin are associated with the synergistic cytotoxicity in GSC11 cells but there is intertumoral heterogeneity in alterations of molecules that are downstream to the targets of the synergistic treatments.

## Discussion

Among several mathematical representations of tumor growth, the Gompertz model has been used in studies of experimental therapies because it has been shown to correlate not only with in vitro cell number and tumor sphere volume but also with in vivo tumor weight and volume in previous studies using conventional cell lines [22, 27, 33–36]. Our study is, as far as we know, the first showing that both number and total volume of colonies of GICs cultured in agarose correlate with the Gompertz function. Correlation of the Gompertz curve with the in vitro tumor growth curves in our study might indicate that tumor growth pattern in this culture system resembles in vivo tumor growth, though the time scale would be different. One of the advantages of 3D culture with gel is that aggregation of cells or colonies can be prevented and thus we can evaluate both accurate colony number that is thought to reflect clonogenicity of GICs and volume of the colonies formed not by cell aggregation but by cell proliferation. In addition, approximately 20–60 colonies are formed in 1 well of 96-well plate that might enable us to analyze the clonogenicity with lower number of wells and plates but higher statistical power than single cell culture system. We found that combination therapy of erlotinib and sorafenib in GSC11 cells was synergistic in both analyses of colony number and total biomass in vitro using the 3D culture method, and that the in vivo combination therapy showed tendency of improvement of survival by the combination therapy, indicating correlation in the therapeutic efficacy between this in vitro assay system and in vivo experimental model. Although we used minimal doses of erlotinib and sorafenib that did not improve the median survival as monotherapies, the combination treatment tended to improve the survival, suggesting supra-additive effect by the combination therapy in vivo. Since the maximum tolerated doses of erlotinib and sorafenib in combination therapy in mouse are higher, efficiency of the combination therapy might be improved by increasing doses of the drugs [37].

Based on the TCGA study that showed possible addiction of glioblastoma cells to several signaling pathways, we evaluated the efficiency of single and combination treatments using RTK inhibitors, non-receptor kinase inhibitors, and transcription factor inhibitors [3]. Since clinical trials of targeted therapies for malignant glioma patients have shown that monotherapies and inappropriate combination therapies have limited efficacy, finding rationale of efficient combination treatments is necessary [4, 8]. This is the first report showing synergistic effects of combinations of EGFR inhibitor with sorafenib, sorafenib with U0126, EGFR inhibitor with MEK inhibitor, and erlotinib with BKM120 on GICs beyond their subtype. Among these synergistic treatments, combination of erlotinib with sorafenib was reported to be synergistic in colorectal and lung cancer cells [37, 38]. Although U0126 was reported to be protective against sorafenib-induced cytotoxicity in hepatocellular carcinoma (HCC) cells, combination of sorafenib with another MEK inhibitor CI-1040 was synergistic associated with increased Bim in HCC cells [39, 40]. Although synergistic effect by erlotinib and U0126 has not been reported, a MEK inhibitor was shown to decrease resistance of non-small cell lung cancer (NSCLC) cells to EGFR inhibitors [41]. Similarly, although synergistic effect by erlotinib and BKM120 has not been reported, combinations of erlotinib and PI3K/mTOR inhibitors showed synergistic cytotoxicity in cells of prostate cancer, NSCLC and ovarian cancer in previous studies [42–44]. Findings in these sporadic studies are consistent with those in our systematic evaluation that show significance of combined inhibition of RTK with PI3K or RAF/MEK. Importantly, inhibition of MEK and PI3K did not induce synergistic effect in our study. Instead, RPPA study indicates that synergistic combination therapy could affect multiple signaling pathways including not only RAS/ERK and PI3K/Akt but also other pathways that are critical for tumor cell survival and proliferation. Therefore, the rationale for the effective combination treatments of GICs might be suppression of both an RTK that activates multiple signaling pathways which form complex signaling network and RAF/MEK or PI3K that could play critical roles in the redundancy of the key signaling pathways.

However, we found significant variations in drug sensitivity and intracellular signaling pathways between GICs. Even GSC2 and GSC20 cells, which are classified into the same subtype of gliomas, showed different sensitivity to most of the mono- and combination therapies. MEK/ERK/NFκB signaling induces translocation of PKM2 into nucleus where PKM2 binds to β-catenin that activates transcription of genes related with cell proliferation including *c*-*Myc* and *CDK1*. [45–47]. Nuclear PKM2 also phosphorylates spindle checkpoint protein Bub3 resulting in mitosis progression [48]. Nuclear β-catenin regulates transcription of *Bcl*-*2* and *Mcl*-*1*. [49]. Therefore, alterations of these molecules in GSC11 cells induced by combination of erlotinib with sorafenib were consistent with the increased cytotoxicity by the combination therapy. However, GSC20 cells responded to combination of erlotinib with sorafenib in a different manner from that of GSC11 cells regarding both cell death (Fig. 3b) and signaling molecules (data not shown). In addition, phosphorylated Akt and ERK in GSC2 cells treated with erlotinib and sorafenib also showed different alteration patterns from those in GSC11 or GSC20 (data not shown). Given that intratumoral heterogeneity contributes to therapy resistance and recurrence of the tumor in a patient, our findings indicate that intertumoral heterogeneity of GICs in sensitivity to cytotoxic stimuli associated with diverse alterations in intracellular signaling pathways could be another reason for the failure of the “one-size-fit-all” designed treatment for malignant glioma patients [5, 8, 50]. In several types of cancers, novel classifications based on biomarkers or profiling are providing actionable and critical information to make clinical decisions [51]. In glioma patients, there is a possibility that a small number of molecular features such as CpG island methylation phenotype (G-CIMP) or IDH1 mutation status could be useful for prediction of the outcome [32, 52]. In addition to these factors, different status of several key molecules could alter dependency of GICs on the key signaling pathways or crosstalk/feedback network [3, 14, 28, 29, 41, 53]. In this study, we found that at least GSC2, GSC11 and GSC20 are neither G-CIMP nor IDH1 mutant subtype (data not shown) and have different gene expression patterns regarding RTKs, PI3K, RAS and Rb pathways that could induce the diversity in the drug sensitivity of the GICs even in the same subtype (Additional file 3). However, our findings as well as previous studies indicate difficulty in predicting sensitivity of glioma cells to specific treatment by alterations in small number of genes or proteins [30, 54]. These findings have been fueling profiling approach using large data set [19, 32, 55]. However, the current subtype classification of gliomas might not be sufficient for selecting the best treatment for patients while certain extent of the effectiveness was found [19]. Therefore, it is necessary to establish a more effective personalized optimization method for glioma therapy. Although hypothesis-driven studies with omic analyses that attempt to predict sensitivity of tumors are intensely performed, unpredictability of cancer cell response to specific treatment has not yet been overcome. Empiric approaches such as treatment sensitivity testing using cultured tumor cells obtained from each patient have also been studied but have not yet significantly improved outcome of glioma patients [56, 57]. In this study, we showed that synergistic combination treatments for different cell lines with diverse drug sensitivity can be efficiently identified by examining theory-driven combinations, which are RTK inhibitors and RAF/MEK or PI3K inhibitors, indicating that integration of rationale-based candidate selection with empiric screening approach is useful to optimize therapy for individual patients against intertumoral heterogeneity.

### Role of autophagy in efficient combination therapy

Among the synergistic combination treatments, we focused on the combination of erlotinib and sorafenib because clinical trials for patients with tumors including malignant gliomas to examine efficacy of the combination therapy were ongoing when we started this study [50, 58]. This is the first report showing autophagic cell death induced by the combination treatment. Autophagy is thought to be a cellular process for homeostasis and adaptation to stress and is thus cytoprotective mechanism [59]. However, unregulated excessive autophagy is known to be cytotoxic, inducing autophagic cell death [59]. Results in this study indicate that monotherapy of erlotinib might induce cytoprotective autophagy but the combination of erlotinib and sorafenib causes cell-killing autophagy. Autophagy might be associated with cellular senescence whose role in antitumor therapy has not yet been established because the senescence in tumor cells can contribute to not only therapy resistance but also growth suppression or sensitization to therapy [60–62]. While these findings suggest the complex role of autophagy in tumor therapy, our observation might indicate the potential of combination therapies that induce autophagy-related remarkable cytotoxicity in tumor-initiating cells.

## Conclusion

We found that growth of GICs in agarose gel correlates with the Gompertz function. Combination treatments that affect RTKs and the proximal downstream molecules RAF/MEK or PI3K induce synergistic cytotoxicity in different GICs that show diversity in drug sensitivity. Among these combination treatments, combination of erlotinib with sorafenib induces apoptotic and autophagic cell death associated with attenuated Akt and ERK signaling, decreased nuclear PKM2 and β-catenin and affected Bcl-2 family proteins in GSC11 cells in vitro, and tends to improve survival of the brain tumor xenografts. Although sensitivity of GIC lines to anticancer agents is diverse, the rationale for efficient combination therapy is thought to be inhibition of both RTK and RAF/MEK or PI3K. This rationale and the method in this study could be useful for personalized drug screening using GICs from each patient to optimize targeted therapy for glioma patients.

## Additional files

10.1186/s12967-016-0803-2 Supplementary tables.
10.1186/s12967-016-0803-2 Supplementary figure.
10.1186/s12967-016-0803-2 Gene expression of GSC11, GSC2 and GSC20.
10.1186/s12967-016-0803-2 Antibody list in RPPA analysis.
10.1186/s12967-016-0803-2 Data of RPPA analysis.
