# Supplementary material for: Suppression of RAF/MEK or PI3K synergizes cytotoxicity of receptor tyrosine kinase inhibitors in glioma tumor-initiating cells
Source: J Transl Med. 2016 Feb 9;14:46. doi: 10.1186/s12967-016-0803-2 (PMC4746796; doi:10.1186/s12967-016-0803-2)
Supplement: Supplementary file 1 — 10.1186/s12967-016-0803-2 Supplementary tables. [file 12967_2016_803_MOESM1_ESM.doc]

**Supplementary table 1. Gomperzian analysis^*^ of total biomass and colony number of glioma tumor-initiating cells and cell lines cultured in agarose**

Cell line A α V_0_

Total biomass

GSC11 1.0874 +/- 0.1296 0.1720 +/- 0.02035 3154 +/- 1408

GSC20 0.9089 +/- 0.1256 0.1195 +/- 0.01229 2755 +/- 1139

U87 1.601 +/- 0.2170 0.1731 +/- 0.01081 1171 +/- 1110

LNZ308 0.7133 +/- 0.1594 0.1069 +/- 0.02061 934.8 +/- 460.8

LN428 1.943 +/- 0.3200 0.3067 +/- 0.03190 3310 +/- 1937

Colony number

GSC11 1.4679 +/- 0.1915 0.3387 +/- 0.02935 2.9 +/- 1.5

GSC20 1.2138 +/- 0.2769 0.2486 +/- 0.04509 2.7 +/- 1.1

U87 1.7900 +/- 0.1064 0.3228 +/- 0.05201 0.83 +/- 0.3267

LNZ308 0.8173 +/- 0.2419 0.1761 +/- 0.04621 0.83 +/- 0.65

LN428 2.348 +/- 0.4047 0.4775 +/- 0.03112 2.7 +/- 1.6

(A/α)

(1-e^-αt^)

^*^; Gompertzian equation:

V = V_0_ x e

where A and α are constants, V_0_ is the total volume (μm^3^) or the number of the colonies at time zero, and V is the total volume (μm^3^) or the number of the colonies at time t. Data are means +/- 95% confidence intervals of at least three independent experiments.

**Supplementary Table 2. Non-synergistic combinations in glioma tumor-initiating cells**

Drugs GSC11 GSC20 GSC2

Erlotinib + Imatinib 0.98 +/- 0.41 1.97 +/- 0.89 1.86 +/- 0.04

Erlotinib + PP2 1.08 +/- 0.20 1.30 +/- 0.04 1.13 +/- 0.21

Erlotinib + Myc II^A^ 1.34 +/- 0.75 1.03 +/- 0.20 1.12 +/- 0.52

Erlotinib + WP1066 1.17 +/- 0.45 1.07 +/- 0.01 1.15 +/- 0.28

Erlotinib + BBR3610 1.04 +/- 0.36 1.09 +/- 0.32 1.38 +/- 0.33

Sorafenib + BKM120 1.60 +/- 0.34

Sorafenib + PP2 1.76 +/- 0.48

Sorafenib + Myc II ^A^ 1.47 +/- 0.20

Sorafenib + WP1066 1.50 +/- 1.12

Sorafenib + BBR3610 1.43 +/- 0.33

U0126 + BKM120 1.14 +/- 0.29 1.13 +/- 0.28 1.20 +/- 0.75

U0126 + PP2 2.03 +/- 0.84

U0126 + Myc II ^A^ 1.47 +/- 0.65

U0126 + WP1066 1.52 +/- 0.59

Chou and Talalay combination indices (CI) are shown as means +/- 95% confidence intervals of at least two independent experiments. According to the original study by Chou and Talalay, CI < 1, = 1; and > 1 indicate synergistic, additive, and antagonistic, respectively. In this study, combination effect was thought to be synergistic when CI was less than 0.9. ^A^; Myc II = c-Myc inhibitor II.

**Supplementary Table 3. Combination effects of erlotinib and sorafenib on tumor sphere growth of GSC11 and GSC20 cells in agarose**

Chou-Talalay Tumor growth delay

Combination index erlotinib sorafenib combination

GSC11 0.45 +/- 0.13 0.19 0.032 1.45

GSC20 0.94 +/- 0.03 0.20 0.45 0.85

Data of combination index (CI) shown are means +/- 95% confidence intervals of three independent experiments. Data of tumor growth delay (TGD) are representative data of three independent experiments. According to the original study by Chou and Talalay, CI < 1, = 1; and > 1 indicate synergistic, additive, and antagonistic, respectively. In this study, combination effect was thought to be synergistic when CI was less than 0.9. In TGD analysis, combination effect was thought to be synergistic when TGD in combination therapy was larger than summation of TGDs in monotherapies.

**Supplementary table 4. Synergistic effects of erlotinib and sorafenib on signaling pathway proteins in GSC11 cells.**

Protein p-value

X14.3.3.epsilon 0.0308

Akt.pS473 0.001

Akt.pT308 0.0289

C.Raf.pS338 0.0003

MAPK.pT202.Y204 0.0067

p70S6K.pT389 0.0201

PRAS40.pT246 0.0069

S6.pS235.S236. 0.0031

S6.pS240.S244 0.0298

Among 171 proteins analyzed by reverse phase protein array (supplementary data), proteins on which the combination treatment induced a statistically significant synergistic effect are listed. pS, pT and pY mean phosphorylated serine, threonine, and tyrosine, respectively.
