# Supplementary figures and images for: Suppression of RAF/MEK or PI3K synergizes cytotoxicity of receptor tyrosine kinase inhibitors in glioma tumor-initiating cells
Source: J Transl Med. 2016 Feb 9;14:46. doi: 10.1186/s12967-016-0803-2 (PMC4746796; doi:10.1186/s12967-016-0803-2)

# Supplementary figure 1

**A**

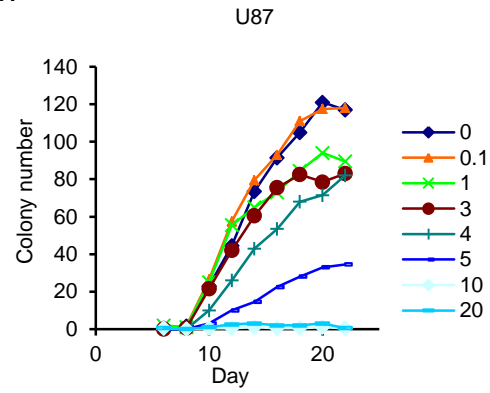

**B**

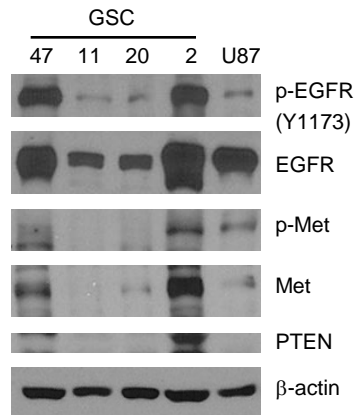

Supplement: Supplementary file 2 — 10.1186/s12967-016-0803-2 Supplementary figure. [file 12967_2016_803_MOESM2_ESM.pdf]
